# Impact of biodiversity-climate futures on primary production and metabolism in a model benthic estuarine system

**DOI:** 10.1186/1472-6785-11-7

**Published:** 2011-02-14

**Authors:** Natalie Hicks, Mark T Bulling, Martin Solan, Dave Raffaelli, Piran CL White, David M Paterson

**Affiliations:** 1 Sediment Ecology Research Group, Scottish Oceans Institute, University of St Andrews, East Sands, St. Andrews, Fife, KY16 8LB, UK; 2 Oceanlab, University of Aberdeen, Main Street, Newburgh, Aberdeenshire, AB41 6AA, UK; 3 Environment Department, University of York, Heslington, York, YO10 5DD, UK

## Abstract

**Background:**

Understanding the effects of anthropogenically-driven changes in global temperature, atmospheric carbon dioxide and biodiversity on the functionality of marine ecosystems is crucial for predicting and managing the associated impacts. Coastal ecosystems are important sources of carbon (primary production) to shelf waters and play a vital role in global nutrient cycling. These systems are especially vulnerable to the effects of human activities and will be the first areas impacted by rising sea levels. Within these coastal ecosystems, microalgal assemblages (microphytobenthos: MPB) are vital for autochthonous carbon fixation. The level of *in situ *production by MPB mediates the net carbon cycling of transitional ecosystems between net heterotrophic or autotrophic metabolism. In this study, we examine the interactive effects of elevated atmospheric CO_2 _concentrations (370, 600, and 1000 ppmv), temperature (6°C, 12°C, and 18°C) and invertebrate biodiversity on MPB biomass in experimental systems. We assembled communities of three common grazing invertebrates (*Hydrobia ulvae, Corophium volutator *and *Hediste diversicolor) *in monoculture and in all possible multispecies combinations. This experimental design specifically addresses interactions between the selected climate change variables and any ecological consequences caused by changes in species composition or richness.

**Results:**

The effects of elevated CO_2 _concentration, temperature and invertebrate diversity were not additive, rather they interacted to determine MPB biomass, and overall this effect was negative. Diversity effects were underpinned by strong species composition effects, illustrating the importance of individual species identity.

**Conclusions:**

Overall, our findings suggest that in natural systems, the complex interactions between changing environmental conditions and any associated changes in invertebrate assemblage structure are likely to reduce MPB biomass. Furthermore, these effects would be sufficient to affect the net metabolic balance of the coastal ecosystem, with important implications for system ecology and sustainable exploitation.

## Background

Rising global temperatures and increasing atmospheric CO_2 _concentrations are causing changes to a wide range of ecosystems [1]. The influence of these changing conditions on ocean chemistry and the distribution of species in marine systems [2-4] is of particular concern. Atmospheric concentrations of CO_2 _have risen from pre-industrial levels (275 ppmv) to 370 ppmv and continue to increase by ~1.5 ppmv yr^-1 ^[5]. Up to 50% of the global increase in carbon dioxide has been absorbed by the oceans [6] and the pH of the sea is predicted to fall by up to 0.5 pH units by the end of the century [2]. Concerns over the likely consequences are now widespread [7,8]. Studies of elevated carbon dioxide concentrations have demonstrated potential impacts on nutrient availability, primary productivity and decomposition [1,9,10]. This directly influences the functionality of ecosystems [9] across multiple trophic levels [11] and effects are difficult to anticipate.

Empirical research to date has concentrated on the responses of a variety of ecosystems to individual anthropogenic drivers of change (terrestrial [12]; marine [13]; terrestrial soil [14]; freshwater [9]), and few studies have considered the combined effects of multiple drivers [15]. This is of particular importance because cumulative and/or interactive effects between drivers are very likely to be influential in determining levels of ecosystem functioning [10,16-22]. It is well recognised that services derived from ecosystems are essential to human welfare [15,23-26] and could be critically affected through climate change [26,27]. Consequently, research that examines the effects of multiple climate change factors, such as temperature and carbon dioxide, and altered levels of biodiversity on ecosystem functioning is essential and timely.

In the face of growing concern about climate change, the net carbon status of many coastal and estuarine systems has received increasing attention [28]. Net allochthonous systems rely on an external carbon supply for the majority of their carbon metabolism (heterotrophic) while autochthonous systems are dominated by *in situ *carbon fixation (autotrophic). This distinction has been widely applied in the study of lotic systems (e.g. the river continuum concept [29]) and has recently been applied to the metabolic status of coastal systems to understand their potential to respond to exploitation [30,31]. Relatively subtle environmental perturbations may alter the balance between autotrophy and heterotrophy [32] having profound effects on these ecosystems and the organisms that exploit them. MPB are the main primary producers in many intertidal and shallow subtidal depositional environments [33], and enhance benthic-pelagic coupling through the formation of biofilms [34,35]. Our hypothesis was that interactions between climate change variables and biodiversity would inhibit autochthonous productivity by MPB [36-38] and hence would affect the net trophic status of these vulnerable coastal systems. This is because CO_2 _levels *per se *do not appear to enhance MPB photosynthesis, while acidification and grazing activity both have a negative influence. In this paper, we examine the effects of two climate change variables (temperature and atmospheric CO_2 _concentration) within the context of a range of biodiversity levels (macrofaunal species richness) on the biomass of MPB using a model multi-trophic level [39] experimental mesocosm system.

## Methods

### Sediment

Surface sediment (< 2 cm depth) was collected from tidal mud flats on the Ythan Estuary, Aberdeenshire, Scotland, UK (57°20.085'N, 02°0.206' W) and sieved (500 μm) in a seawater bath (UV sterilised, 10 μm filtered, salinity 33 psu) to remove macrofauna. The sediment was left to settle for 48 h before the supernatant was removed, the sediment homogenised and placed in the mesocosms to a depth of 10 cm (785 cm^3^).

### Microphytobenthos

To standardize the biomass of the MPB, MPB-rich surface sediment was collected from the Ythan estuary, spread onto a shallow tray (< 1 cm depth) and left under constant light for 48 h. This material was then homogenised and distributed (125 cm^3 ^aliquots) between mesocosms prior to the addition of seawater.

### Macrofauna

The polychaete *Hediste (Nereis) diversicolor *(HD), the gastropod *Hydrobia ulvae *(HU) and the amphipod *Corophium volutator *(CV) were collected from the study site. These species represent a range of functional types in the way that they bioturbate sediments, and hence drive nutrient flux [40,41], and their mode of grazing on MPB [42].

Replicate (n = 3) macrofaunal communities were assembled in single and multispecies treatments (HD, HU, CV, HDHU, HDCV, HUCV, HDHUCV). These unique species permutations eliminate pseudoreplication [43] and allow the generic effects of altered biodiversity to be examined. Macrofaunal biomass was set at 2 g wet weight per mesocosm (divided equally between the species present), similar to the natural biomass found at the study site [44]. Control mesocosms (n = 3) containing the standard MPB biomass but without any macrofauna were also established. There were a total of 24 mesocosms per environmental chamber (see supporting information in Additional file 1).

### Mesocosm structure and assembly

Mesocosms were Perspex cores 33 cm high with an internal diameter of 10 cm. Following the addition of sediment (10 cm deep) to each mesocosm, 125 cm^3 ^of MPB- rich sediment and 2.35 l of seawater (UV-sterilized, 10 μm pre-filtered, salinity ≈ 33) were added to give an overlying depth of 20 cm. This initial fill of water was replaced after 24 h to remove the nutrient pulse associated with assembly [40] and macrofauna were then added. All mesocosms were non-tidal and were aerated individually throughout the experiment with the defined CO_2 _atmospheric level within the chamber. A total of 216 mesocosms were required.

### Environmental regimes

Mesocosms were placed in two environmental chambers (24 per chamber per run, V 4100, Vötsch Industrietechnik) with temperature control (± 0.1°C). The experiments were run with a 12 h light - 12 h dark (L/D) cycle using high intensity discharge sodium lamps (model GE11678, 400 w ×2, average 300 μmoles m^-2 ^s^-1^). Nine environmental regimes were employed, using three constant temperatures (6°C, 12°C, and 18°C, reflecting the annual variation at the study site (Additional file 1, Figures S1 - S4)) and three atmospheric carbon dioxide concentrations (370 ppmv (present day), 600 ppmv, 1000 ppmv) in an orthogonal design (see supporting information in Additional file 1). Concentrations of 600 ppmv and 1000 ppmv were based on IPCC projections for approximately 50 and 100 years time respectively [7] and reflect the accepted view that CO_2 _levels will rise over the long-term. Atmospheric CO_2 _concentrations were maintained using a CO_2 _monitor attached to an external CO_2 _gas cylinder (BOC gases Ltd, UK) with a digital controller (Technics horticultural carbon dioxide controller). An Infra-Red Gas Analyser (IRGA, ADC LCA3) was used to calibrate and validate the CO_2 _regulation (± 30 ppm). Mesocosms within an environmental chamber were randomly assigned positions to factor out any effects of spatial heterogenity. Each experiment was run for 7 days.

### PAM fluorescence

The biomass of the MPB was measured at the end of the experiment using a PAM fluorometer (DIVING-PAM, Heinz-Walz GmbH). This is a widely accepted proxy method for measuring the surfice biomass (chlorophyll *a*) of MPB [45,46]. Fluorescence measurements were reported as F_0_^-15 ^^. ^This indicates that the minimum fluorescence was determined after 15 minutes of dark adaptation [47-49]. This time period is a compromise between the time required in MPB to stabilise ubiquinone oxidation, but not so long that surface biomass is altered [45] by cell migration. Three measurements were taken per mesocosm.

### Data Analysis

MPB biomass was treated as a response variable, with macrofaunal species richness (or species combination), CO_2 _concentration and temperature as nominal explanatory variables. Initially, a linear regression model was fitted and assessed for normality (Q-Q plots), homogeneity of variance, and outlying values (Cook's distance) [50,51]. As our experimental design established a gradient of species richness that increased within a finite species pool, variation across treatments was likely to be unequal [40], and this was confirmed by plots of the model residuals. To account for this heterogeneity of variance, a generalised least squares (GLS) [50-52] mixed modelling approach was used in preference to a linear regression of transformed data [10,42,53]. The most appropriate variance-covariate structure for each model was determined using a combination of AIC scores and the examination of plots of fitted values versus residuals based on a full model specification using restricted maximum likelihood (REML) [52]. The minimum adequate model was then determined through manual backwards stepwise selection, using maximum likelihood methods. The significance of the relevant highest order interaction terms was assessed at each stage, terms nested within these not being tested, following Underwood [54]. The influence of each independent term within the minimum adequate model was assessed using a likelihood ratio test (L-ratio) between the minimum adequate model and reduced models (with all terms involving the relevant independent factor removed, including interactions). The L-ratio can be used to assess the order of importance of the independent terms. All analyses were performed using the 'nlme' package (ver. 3.1) [55] in the 'R' statistical and programming environment [56].

## Results

### Microphytobenthos response to climatic variables

**Figure 1:**
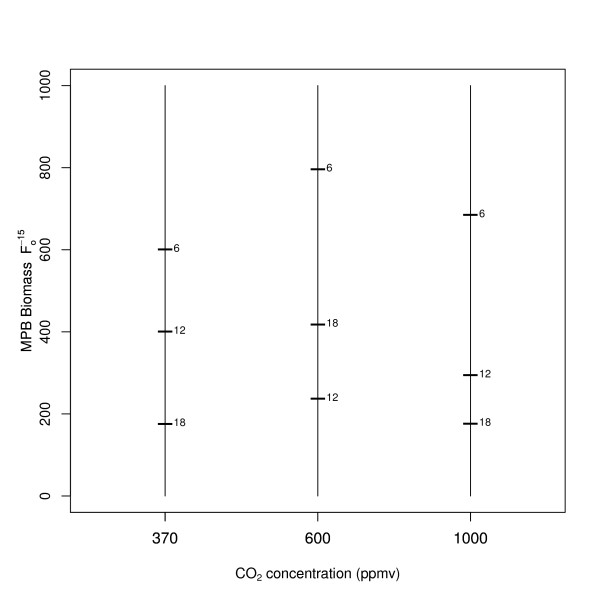
**Model visualisation of temperature and CO_2 _on MPB biomass using only controls (no macrofauna)**. The interaction of temperature (increasing along the x-axis) and CO_2 _(the horizontal bars on each temperature level) on MPB biomass (F_0_^-15^).

The minimal adequate model for the controls (containing no macrofauna), with MPB biomass as the dependent variable, included a two-way interaction (CO_2 _× temperature; *L*-ratio = 18.23, d.f. = 12, *p *= 0.0011). Of the two climatic variables, temperature (*L*-ratio = 37.71, d.f. = 6, *p *< 0.0001) was more influential than CO_2 _(*L*-ratio = 24.51, d.f. = 6, *p *< 0.0001). There was an apparent decline in MPB biomass with increasing temperature (Figure 1 and Additional file 1, Figure S5), whilst the CO_2 _concentration of 600 ppmv was associated with the higher MPB biomass levels, particularly at 6°C and 18°C. This trend was reflected in the model visualisation (Figure 1), with MPB biomass highest at 6°C across all CO_2 _levels.

### Microphytobenthos response to climatic variables and macrofaunal species richness

**Figure 2:**
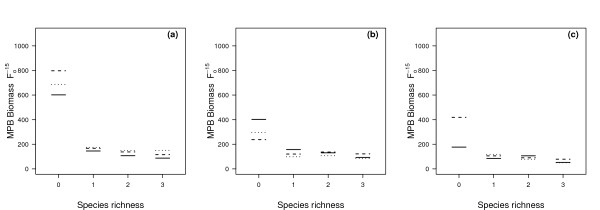
**Model visualisation of the three-way interaction (species richness × temperature × CO_2_) on MPB biomass**. The interaction of temperature, CO_2 _and species richness on MPB biomass (F_0_^-15^) at three constant temperatures (a) 6°C, (b) 12°C, (c) 18°C. In the visualisation, the CO_2 _levels are represented as present-day 370 ppmv (solid line), 600 ppmv (dashed line) and 1000 ppmv (dotted line).

A regression analysis was performed, treating MPB biomass as the dependent variable with the climate variables and macrofaunal species richness as independent variables. The minimal adequate model comprised a three-way interaction (species richness × temperature × CO_2_; *L*-ratio = 23.37, d.f. = 48, *p *= 0.02). Species richness was the most influential variable (*L*-ratio = 95.81, d.f. = 27, *p *< 0.0001), followed by temperature (*L*-ratio = 79.18, d.f. = 24, *p *= 0.0051), and CO_2 _concentration (*L*-ratio = 45.48, d.f. = 24, *p *< 0.0001). The effect of species richness on MPB biomass was most apparent at 6°C (Figure 2 and Additional file 1, Figure S6). Biomass was highest in the absence of macrofauna, and the greatest biomass levels in all three carbon dioxide treatments occurred at the lowest temperature treatment of 6°C.

For both elevated carbon dioxide levels at 6°C, there was an increase in MPB biomass across all species richness levels. This general effect of elevated CO_2 _was not found at the higher temperatures, and overall MPB biomass decreased with rising temperature (Figure 2b, c).

### Microphytobenthos response to climatic variables and macrofaunal species composition

**Figure 3:**
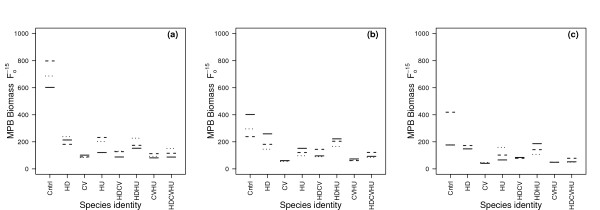
**Model visualisation of the three-way interaction (species identity × temperature × CO_2_) on MPB biomass**. The interaction of temperature, CO_2 _and species richness on MPB biomass (F_0_^-15^) at three constant temperatures (a) 6°C, (b) 12°C, (c) 18°C. In the visualisation, the CO_2 _levels are represented as present-day 370 ppmv (solid line), 600 ppmv (dashed line) and 1000 ppmv (dotted line).

A further regression analysis was performed, replacing the explanatory variable species richness with species composition, to determine whether compositional effects underpinned the observed effects of biodiversity. The minimal adequate model comprised a three-way interaction (species composition × temperature × CO_2_; *L*-ratio = 65.52, d.f. = 96, *p *< 0.0001). Species composition was the most influential variable (*L*-ratio = 314.52, d.f. = 63, *p *< 0.0001), followed by temperature (*L*-ratio = 177.46, d.f. = 48, *p *< 0.0001), and CO_2 _(*L*-ratio = 101.26, d.f. = 48, *p *< 0.0001). Increased species diversity had a detrimental effect on MPB biomass, but the magnitude of these effects was dependent on the composition of the invertebrate assemblage within each treatment (Figure 3 and Additional file 1, Figure S7). *C. volutator *had a greater negative effect on MPB biomass than the other macrofaunal species in single species treatments, and the presence of *C. volutator *was a dominant component in the effects of the multispecies treatments (Figure 3). This dominant effect of *C. volutator *decreasing MPB biomass was consistent under all environmental regimes. Importantly, the interaction of the climate drivers mediated the relationship between MPB biomass and species composition, although this mediation was less pronounced in treatments with C. *volutator*, and appeared to have no consistent pattern.

## Discussion

Few ecological studies have examined the effect of multiple simultaneous stressors on individual species and ecosystems [19]. The statistical approach used here does not allow the separation of individual stressor effects, although some inference in terms of the most critical factors can be made from the model metrics (L-ratios) and from the model visualisations. We found complex interactions between the effects of temperature, CO_2 _concentration and macrofaunal species richness/species assemblage composition on ecosystem response, measured as MPB biomass. The MPB biomass provides a proxy estimate of the productive potential of mudflat systems [57] and has been used to model net system productivity. Thus, changes in MPB biomass are a crucial element of system performance and will have reverberating effects through the higher trophic levels [8,38], just as changes in infaunal diversity will affect resource utilisation [58].

In the present study, MPB biomass was higher at low temperature across all CO_2 _regimes, and did not increase as CO_2 _increased, indicating that CO_2 _was not limiting to MPB under these conditions and that increasing temperature was detrimental. The models suggested that species richness/composition and temperature were more influential on MPB than CO_2_, but the interactions between the explanatory variables were significant. The interactive effects of species composition and temperature led to a significant reduction in MPB biomass. The implication of this for temperate estuaries may be quite profound. The overall metabolic balance of estuarine systems between net autotrophy or heterotrophy is under debate [28,36] but the role of autotrophic production by MPB is clear. Reduction of this contribution to the carbon balance will push the system toward a more heterotrophic condition. A shift from autotrophic to heterotrophic conditions, or a shift to more extreme heterotrophy, is likely to have significant, but as yet undetermined, implications for ecosystem goods and services. Unless the net import of allochthonous carbon changes, then overall productivity may be expected to decline with potential effects on resource utilisation. Variation in temperature has already been shown to affect the carbon metabolism of coastal systems [59] and this supports our hypothesis that autochthonous productivity may be reduced through interaction between the climate change variables and species diversity.

In this study, the presence of macrofaunal species resulted in substantial decreases in MBP biomass, and there appeared to be a general decline in MPB with increasing macrofaunal species richness, specifically at the lowest temperature. This general trend is partly to be expected as all three macrofaunal species are known consumers of MPB [34,60]. However, the presence of *C. volutator *had a disproportionately strong effect in reducing MPB biomass, consistent with previous research [34,42,61]. Rather than being attributed to consumption, the mechanism for this dominant effect is likely to be due to the constant resuspension of sediment [42,62] during grazing and bioturbation (Additional file 1, Figure S8), leading to inhibition of photosynthesis by MPB, and also the prevention of MPB biofilm formation on the sediment surface [63].

Individual species responses to climate change are often highly uncertain [64], and environmental change could alter the balance between the functional groups present (through extinction, invasion or changes in abundance or behaviour), as well as the number and identity of species present in an ecosystem [65]. This makes it very difficult to predict how an ecosystem may respond based on diversity alone [66]. MPB utilise nutrients from the water column and pore waters whilst bioturbation by invertebrates is known to increase the flux rates and concentrations (NH_4_-N, PO_4_-P) available [10]. Therefore, species-specific responses to climate change will affect more than one trophic level, and the nature of interactions between species will change as a consequence [8,11,20]. In the present study, the decline in MPB was driven by complex interactions between environmental variables and diversity effects, and mediated through infaunal grazing activity. In this case, there was no apparent compensation through an increase in nutrients caused by bioturbatory activity. The functional importance of species is also context-dependent, and functional impact may alter as conditions change. Thus, while functional diversity is important, it may be overshadowed by species identity as different species take up more prominent roles under changing scenarios. In our experiment, the dominant effect of one species (*C. volutator*) illustrates how the extinction of influential (rare or common) species may have profound effects on the ecosystem - and that these effects may be direct or indirect [67,68].

Many studies focusing on the ecological consequences of altered biodiversity within the context of specific drivers of environmental change have concentrated on single variables and few species [11,64,65,69]. Whilst these studies are informative in understanding the mechanisms behind ecosystem response, care must be taken in making predictions based on simplistic assumptions, such as additive and linear relationships. Studying the next level of complexity is problematic, and while a mesocosm approach may help provide conceptual advances, we recognise the limitations of any artificial system in providing realistic interpretations of natural ecosystem response [70]. However, we have shown that interactive effects can have a fundamental influence on MPB biomass and since the balance between autotrophic and heterotrophic status in transitional systems may be delicate [27], then there is a real possibility that climate change may force an overall change metabolism in coastal systems. Given that coastal systems will be at the forefront of climate change effects, they may undergo profound changes in the near future with associated implications for ecosystem services.

## Conclusions

The interactive effects between climate drivers and other anthropogenic factors, such as pollution, habitat destruction and overfishing [20,69,71] add further difficulty to any interpretation of climate change effects. However, there is an urgent need to provide relevant scientific results as guidance for effective action in response to climate change. This requires recognition of the complex interactions between the chemical, physical and biological components of an ecosystem [22,72-74] through a combination of empirical studies, long-term observational data and ecological modelling [64,72,75]. Such data will allow us to gain a greater understanding of the link between ecosystems and the services we obtain from them, levels of variation in ecosystem structure and functioning, and data on how these systems are changing within the context of multiple anthropogenic drivers.

## Authors' contributions

NH carried out the experimental procedures, coordinated the study, performed the statistical analysis and drafted the manuscript. MTB assisted in all experimental procedures, provided statistical support and helped draft the manuscript. MS and DMP participated in the design and coordination of the study, and assisted interpretation of statistical analysis. DR and PCLW conceived initial ideas for the study. All authors read, edited and approved the final manuscript.

## Supplementary Material

Additional file 1 **Supporting Information**. •Additional graphs of raw data, summary of study site temperatures, final models in full, figures referred to in the text as Figures S, schematics of experimental set up and design. •Supporting material for the data presented in the main body of the paper, including annual temperature graph of study site, supplementary figures referred to in the text, box plots of raw data, bootstrapping of models, and full final models. Click here for file
