# Supplementary material for: Impact of biodiversity-climate futures on primary production and metabolism in a model benthic estuarine system
Source: BMC Ecol. 2011 Feb 14;11:7. doi: 10.1186/1472-6785-11-7 (PMC3046901; doi:10.1186/1472-6785-11-7)
Supplement: Additional file 1 — Supporting Information. •Additional graphs of raw data, summary of study site temperatures, final models in full, figures referred to in the text as Figures S, schematics of experimental set up and design. •Supporting material for the data presented in the main body of the paper, including annual temperature graph of study site, supplementary figures referred to in the text, box plots of raw data, bootstrapping of models, and full final models. [file 1472-6785-11-7-S1.DOC]

**Supplementary Material**

**Impact of biodiversity-climate futures on primary production and metabolism in a model benthic estuarine system**

Natalie Hicks*^1,2^, Mark T. Bulling^2,3^, Martin Solan^2^, Dave Raffaelli^3^, Piran C. L. White^3^ and David M. Paterson^1^

*^1^ Sediment Ecology Research Group, Scottish Oceans Institute , University of St Andrews, East Sands, St. Andrews, Fife, KY16 8LB, United Kingdom*

*^2^ Oceanlab, University of Aberdeen, Main Street, Newburgh, Aberdeenshire, AB41 6AA, United Kingdom*

*^3^ Environment Department, University of York, Heslington, York YO10 5DD, United Kingdom*

**Correspondence author: Tel: + 44 1224 274401; Fax: + 44 1224 274402;*

*email:* [*natalie.hicks.50@aberdeen.ac.uk*](mailto:natalie.hicks.50@aberdeen.ac.uk)

*Figure S1: Set up of environmental chambers*

Mesocosms were placed in an environmental chamber (VC 4100, Vötsch Industrietechnik) which maintained a constant temperature environment (± 0.1 ˚C). The atmosphere within the chamber was maintained (± 30 ppm) using a CO_2_ monitor and controller linked to the gas regulator. Regulation of atmospheric CO_2_ was achieved using an infra red gas analyser (ADC, LCA3) and the CO_2_ rich air was bubbled into each individual mesocosm. This follows the experimental set up of Bulling *et al*. 2010.


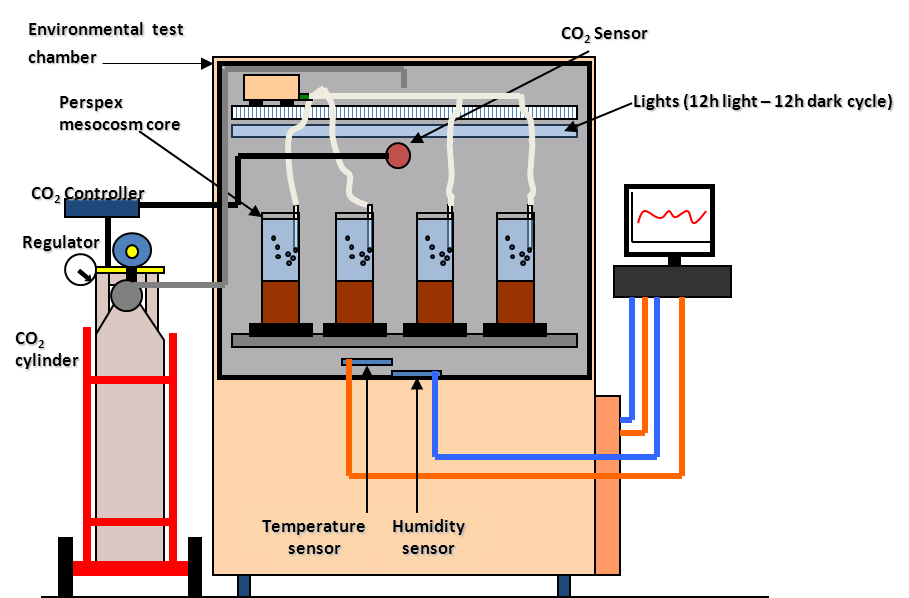


*Figure S2: Experimental design and treatments*

The experiment was run during the late spring and early summer months of 2008, with each successive temperature treatment applied at appropriate times (sequence listed below, upper panel) to match the ambient seasonal water temperature at the study site (see below, lower panel).

*Figure S3: Macrofaunal Species Richness and Composition*

For each experimental run there were three replicates of eight treatments (i.e. n = 24 mesocosms per chamber, depicted below). The controls contained only MPB with no macrofauna.


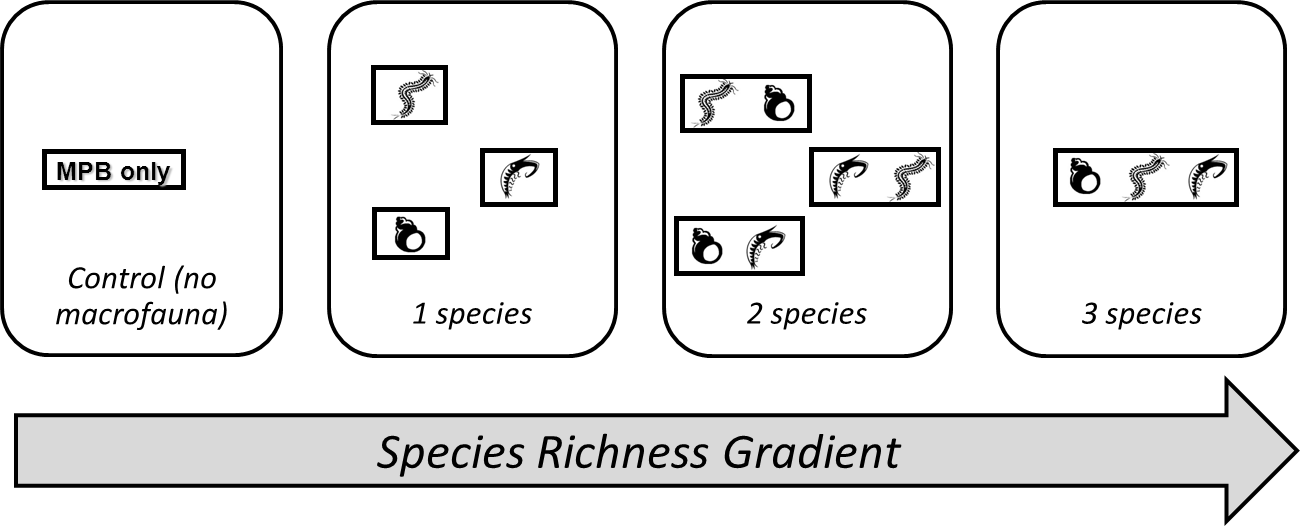


*Figure S4:* *Annual Variation of Water Temperature from Ythan Estuary*

The maximum (solid line) and minimum (dotted line) daily water temperatures from 01/01/2005 to 11/11/2009 recorded in the Ythan estuary, Newburgh, Aberdeenshire

*Figure S5: Boxplots of Raw Data*

Boxplot showing the raw data for mean MPB biomass at 6°C (red), 12°C (green) and 18°C (blue) for each CO_2_ concentration

|  |
| --- |

*Figure S6a: Bootstrapped models with confidence intervals*

Confidence intervals (the error bars) around model predictions were estimated using a bootstrapping methodology (Efron & Tibshirani, 1993). Model residuals were randomly reordered to create an adjusted dataset and the statistical model parameters were recalculated using this adjusted dataset. Model predictions were made for every possible combination of independent variable factor levels. This whole process was repeated 1000 times, to allow generation of bootstrapped distributions around the original model predictions. Bootstrapped models of MPB biomass with increasing Species Richness at 6°C (left column), 12°C (middle column) and 18°C (right column) for each CO_2_ concentration (370 ppmv, top row; 600 ppmv, middle row; 1000 ppmv, bottom row).

*Figure S6b: Boxplots of Raw Data for Species Richness*

Boxplots showing the raw data for MPB biomass with increasing Species Richness at 6°C (left column), 12°C (middle column) and 18°C (right column) for each CO_2_ concentration (370 ppmv, top row; 600 ppmv, middle row; 1000 ppmv, bottom row).

**MPB Biomass (F_o_^-15^)**

**Species Richness**

*Figure S7a: Bootstrapped models with confidence intervals*

Confidence intervals around model predictions were estimated using the bootstrapping methodology as described in Fig. S3a. Bootstrapped models of MPB biomass for each Species Identity at 6°C (left column), 12°C (middle column) and 18°C (right column) for each CO_2_ concentration (370 ppmv, top row; 600 ppmv, middle row; 1000 ppmv, bottom row).

*Figure S7b: Boxplots of Raw Data for Species Identity*

Boxplots showing the raw data for MPB biomass for each Species Identity at 6°C (left column), 12°C (middle column) and 18°C (right column) for each CO_2_ concentration (370 ppmv, top row; 600 ppmv, middle row; 1000 ppmv, bottom row).

**Species Identity**

**MPB Biomass (F_o_^-15^)**

*Figure S8: Image of Sediment Resuspension*

Four sample cores at the end of an experimental run illustrating the effect of activity by *Corophium volutator* in resuspending sediment into the water column. The two on the right contain *C. volutator* whilst the two on the left with clear water contain no *C. volutator*.

| 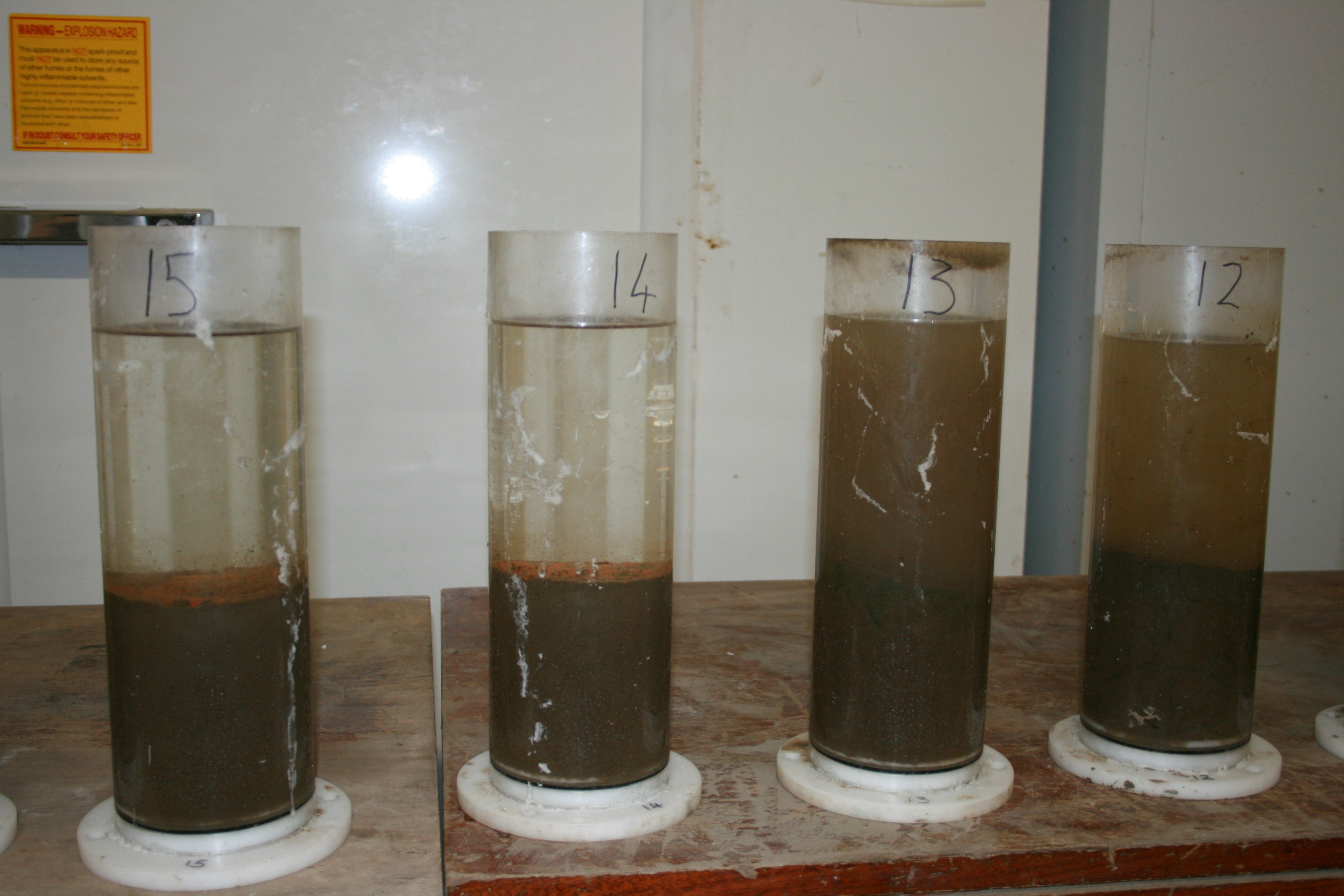 |
| --- |

*Boxplots of raw data of pH measurements within mesocosms*

Boxplots showing the raw data of pH measured within the mesocosms for each of the CO_2_ regimes across an increasing species richness gradient (top graph) and species assemblage composition (bottom graph). The colour of the boxes indicates the CO_2_ regime: green (370 ppmv); orange (600 ppmv); red (1000 ppmv). This shows the pH decreasing with increased atmospheric CO_2_.

*Structure of the minimal adequate models*

**a) Model for MPB biomass with carbon dioxide and temperature as independent variables**

MPB Biomass = f(CO2 conc. + Temperature

+ CO2 conc. × Temperature)

The model was a linear regression model with a gls extension (Temperature variance-covariate).

**b) Model for MPB biomass with carbon dioxide, temperature and species richness as independent variables**

MPB Biomass = f(Species richness + CO2 conc. + Temperature

+ Species richness × CO2 conc.

+ Species richness × Temperature

+ CO2 conc. × Temperature

+ Species richness × CO2 conc. × Temperature)

The model was a linear regression model with a gls extension (Temperature and Species richness variance-covariates).

**c) Model for PO4-P concentrations with species richness as an independent variable.**

MPB Biomass = f(Species identity + CO2 conc. + Temperature

+ Species identity × CO2 conc.

+ Species identity × Temperature

+ CO2 conc. × Temperature

+ Species identity × CO2 conc. × Temperature)

The model was a linear regression model with a gls extension (Temperature and Species identity variance-covariates).

**References**

Bulling MT, Hicks N, Murray LM, Solan M, Raffaelli D, White PCL, Paterson DM: **Marine biodiversity-ecosystem functions under uncertain environmental futures.** *Philosophical Transactions of the Royal Society B* 2010*,* **365**, 2107-2116.

Efron B, Tibshirani RJ: **An Introduction to the Bootstrap.** Chapman and Hall, New York, 1993.

END OF SUPPLEMENTARY MATERIAL
